# Gambogic acid inhibits LPS-induced macrophage pro-inflammatory cytokine production mainly through suppression of the p38 pathway 

**DOI:** 10.22038/IJBMS.2018.23897.5995

**Published:** 2018-07

**Authors:** Jianjun Ma, Kangmao Huang, Yan Ma, Shuai Chen, Chao Liu, Zhi Shan, Xiangqian Fang

**Affiliations:** 1 Department of Orthopaedic Surgery, Sir Run Run Shaw Hospital, Medical College of Zhejiang University, Hangzhou, China; 2 Sir Run Run Shaw Institute of Clinical Medicine of Zhejiang University, Hangzhou, China

**Keywords:** Anti-inflammatory agents, Gambogic acid, MAPK, NF-κB, p38, RAW 264.7 cells

## Abstract

**Objective(s):**

In traditional Chinese medicine, gamboge can detoxify bodies, kill parasites, and act as a hemostatic agent. Recent studies have demonstrated that gambogic acid (GBA) suppressed inflammation in arthritis, and also presented antitumor effect. Thus, this study investigated the new biological properties of GBA on macrophages.

**Materials and Methods:**

RAW 264.7 cells were pretreated with GBA at different concentrations (10, 20, 40, 80, 160, 320 nM) for 24 hrs, and then cell viability was measured using Cell Counting Kit (CCK)-8 assays. Pro-inflammatory cytokines such as TNF-α, IL-6 and IL-1β were determined using ELISA kits and qPCR. Then nitrite concentration was calculated according to a standard curve. At last, the effect of GBA on MAPK and NF-κB signaling pathways was assessed by western blot and luciferase reporter gene assay.

**Results:**

GBA (IC50: 260 nM) suppressed the TNF-α, IL-6 and IL-1β expression induced by lipopolysaccharide (LPS) in RAW 264.7 cells. The expression of TNF-α, IL-6 and IL-1β decreased to 30-50% and 70-75% in the high-dose (160 nM) and low-dose (40 and 80 nM) GBA groups, respectively. Furthermore, the nitric oxide (NO) production and the activation of NF-κB, ERK, and JNK pathways were significantly reduced in high-dose (160 nM) GBA only, while p38 pathway was inhibited at both low (40 and 80 nM) and high (160 nM) concentration of GBA.

**Conclusion:**

These data suggested that GBA inhibited LPS-induced production of pro-inflammatory cytokines including TNF-α, IL-6 and IL-1β mainly through the suppression of the p38 pathway.

## Introduction

Periodontitis is an inflammatory response mainly induced by bacterial pathogens (1, 2). Lipopolysaccharide (LPS) is a key mediator of the inflammatory response and it can stimulate macrophages to release cytokines that promote inflammatory responses, such as tumor necrosis factor-α (TNF-α), interleukin-1β (IL-1β), and interleukin-6 (IL-6) (3). LPS induces the inflammatory reaction in macrophages via the activation of nuclear factor-κB (NF-κB) and mitogen-activated protein kinase (MAPK) pathways, including extracellular signal-regulated kinases (ERK), c-Jun N-terminal kinase (JNK) and p38 pathways (4-7). A previous review reported that the p38 pathway plays a key role in the orchestration of the inflammatory response in macrophages (6) and performs an important function in the control of TNF-α gene expression (6). Thus, the LPS-stimulated RAW 264.7 cells are an excellent model to screen candidate compound targeted in the inflammatory pathways (5). Gambogic acid (GBA) is an active agent extracted from gamboges (8), and has been used as an antitumor compound in China (9, 10). Furthermore, GBA also suppresses inflammation in rats with rheumatoid arthritis and spinal cord injury (11, 12). Thus, we identified GBA as a promising drug for the treatment of patients with periodontal diseases through the inhibition of inflammation. Here, we investigated the mechanism by which GBA inhibits LPS-induced release of pro-inflammatory factors in RAW 264.7 macrophages.

## Materials and Methods

***Tested compounds and chemicals***

Mouse RAW 264.7 cells were purchased from the American Type Culture Collection (Rockville, MD). *Porphyromonas gingivalis* LPS was purchased from InvivoGen (Cayla, France). Alpha modification of Eagles medium (α-MEM) and fetal bovine serum (FBS) were obtained from Gibco-BRL (Sydney, Australia). GBA was purchased from Herbest (Baoji, Shanxi China) and dissolved in dimethylsulfoxide (DMSO). Griess Reagent was purchased from Promega (USA). The Prime Script RT reagent kit and SYBR Premix Ex Taq^TM^ II were purchased from TaKaRa Biotechnology (Otsu, Shiga, Japan). Mouse TNF-α, IL-6 and IL-1 ELISA kits were purchased from Biolegend Co. (San Diego, California, USA). Antibodies against phospho-ERK1/2, ERK1/2, phospho-JNK1/2, JNK1/2, and IκB-α were purchased from Cell Signaling (Danvers, MA). Cell Counting Kit (CCK)-8 kit was obtained from Dojindo Molecular Technology (Kumamoto, Japan). All other chemicals and reagents were purchased from Sigma (USA).

***Cell culture and treatment***

RAW 264.7 cells were cultured in complete α-MEM medium (α-MEM containing 10% heat inactivated FBS and 1% penicillin-streptomycin solution) in a humidified incubator with 5% CO_2_ at 37 ^°^C. The cells were pretreated with different concentrations of GBA and stimulated with LPS (100 ng/ml) for the indicated times. GBA was dissolved in DMSO, and the final concentration of DMSO was less than 0.1%.

***Assay of cell viability***

RAW 264.7 cells were plated in 96-well plates at the density of 2×10^4^ cells/well overnight. The cells were pretreated with GBA at the different concentrations (10, 20, 40, 80, 160, 320 nM) for 24 hrs. Then, 10 μl of CCK-8 buffer was added to each well and the cells were incubated at 37^ °^C for another 2 hrs, after which the absorbance was measured at 450 nm (650 nm reference) on an ELX800 absorbance microplate reader (Bio-Tek, Winooski, VT, USA). Cell viability was calculated relatively to that of the control group from the optical density (OD) by using the following formula: Cell viability = experimental group optimal density OD − reference OD/control group OD – reference OD.

***ELISA for TNF-α, IL-6 and IL-1β***

RAW 264.7 cells were seeded in a 6-well plate with a density of 1×10^6^ cells per well. The cells were pretreated with the indicated concentration of GBA for 2 hrs and then exposed to LPS (100 ng/ml) for 24 hrs. The expression of TNF-α, IL-6 and IL-1β in the supernatants were determined using ELISA kits according to the manufacturer’s instructions. 

***RNA extraction and quantitative real-time PCR (RT-qPCR)***

RAW 264.7 cells were pretreated with the indicated concentration of GBA for 2 hrs and then exposed to LPS (100 ng/ml) for 4 hrs. Total RNA was extracted using Qiagen RNeasy Mini kit (Qiagen, Valencia, CA, USA) according to the manufacturer’s instructions. cDNA was synthesized from 1000 ng of total RNA using reverse transcriptase (TaKaRa Biotechnology, Otsu, Japan). RT-qPCR was performed on an ABI 7500 Sequencing Detection System (Applied Biosystems, Foster City, CA, USA) using the SYBR Premix Ex Taq kit (TaKaRa Biotechnology) with the following cycling program: 40 cycles of denaturation at 95^ °^C for 5 sec and 34 sec of amplification at 60 ^°^C. The following mouse primer sequences were used for RT-qPCR (Table 1): 

***Nitrite determination***

The RAW 264.7 cells (2×104 cells/well) were pretreated with GBA by the fore-mentioned different concentrations for 2 hr then followed by LPS (100 ng/ml) for a further 24 hr to estimate the nitrite concentrations in the supernatant using the Griess Reagent System. For this purpose, 50 µl of supernatant was reacted with 50 µl of sulfanilamide solution and 50 µl of NED solution. Absorbance was measured at a wavelength of 540 nm after incubation for 15 min at room temperature. A standard curve of nitrite concentrations was generated according to the manufacturer’s instructions, and nitrite concentration was calculated using the standard curve.

***NF-κB luciferase reporter gene assays***

To determine whether GBA could affect the NF-κB signaling pathway, RAW 264.7 cells were stably transfected with an NF-κB luciferase reporter construct as previously described (13). Briefly, RAW 264.7 cells were seeded in a 24-well plate at a density of 1×10^5^ cells/well. Cells were pretreated with different concentration of GBA for 2 hrs and then stimulated with LPS (100 ng/ml) for 6 hrs. The luciferase activity was measured using the Promega Luciferase Assay System according to the manufacturer’s instructions.

***Western blot analysis***

RAW 264.7 cells were seeded in a 6-well plate at 1×10^6^ cells/well and pretreated with the indicated concentration of GBA for 2 hrs, followed by LPS (100 ng/ml) stimulation for 30 min (NF-κB and MAPK assays) or 24 hr (inducible nitric oxide synthase, iNOS assay). Western blotting was performed as previously described (14). Membranes were blocked with 5% bovine serum albumin (BSA) for 1 hr and incubated with the indicated primary antibody at 4 ^°^C overnight. After washing three times with Tris-buffered saline with Tween 20 (TBST) buffer, the membranes were incubated with the appropriate secondary antibody for 1 hr at room temperature. The blots were visualized using an Odyssey infrared imaging system (Li-COR).

***Statistical analysis***

All values are presented as the mean ± standard deviation (SD), and each experiment was performed in triplicate. Student’s t-test was used to determine whether there were statistically significant differences between groups. A *P*-value<0.05 was considered as significant difference.

## Results

***The effect of GBA on cells growth and viability***

**Figure 1:**
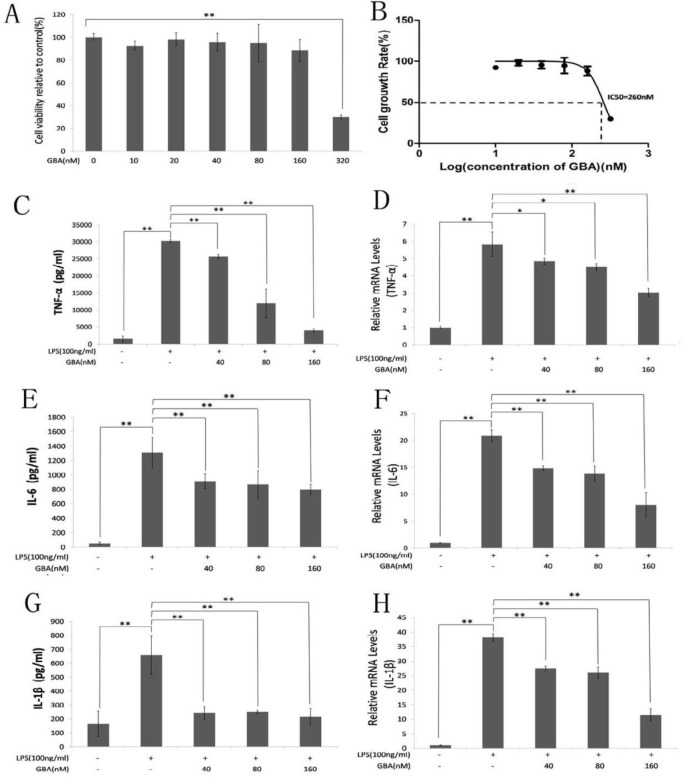
Effect of gambogic acid (GBA) on lipopolysaccharide (LPS)-induced pro-inflammatory cytokine release in raw 264.7 cells. (A) RAW 264.7 cells were pretreated with the indicated concentrations of GBA for 24 hr, and cell viability was measured using CCK-8 assays. (B) The half-maximal inhibitory concentration (IC50) of GBA was 260 nM. (C, E & G) Cells were pretreated with the indicated concentrations of GBA for 2 hr and then exposed to LPS (100 ng/ml) for 24 hr. The expression levels of tumor necrosis factor-α (TNF-α), interleukin-6 (IL-6) and interleukin-1β (IL-1β) in the supernatant were measured by ELISA. (D, F & H) Cells were pretreated with the indicated concentration of GBA for 2 hr and exposed to LPS (100 ng/ml) for 4 hr. The mRNA levels of TNF-α, IL-6 and IL-1β were measured using RT-qPCR. Bars represent the mean±SD of three independent experiments. * *P*<0.01 vs. normal control group; ** *P*<0.01 vs. LPS alone

To assess the effect of GBA on RAW 264.7 cells, we first measured cell viability using a CCK-8 kit. As shown in Figure 1A, cell viability was not affected by GBA within 24 hr at 160 nM of GBA. However, obvious cytotoxicity was observed when RAW 264.7 cells were treated with 320 nM GBA. The calculated IC_50_ of GBA for this cell line was 260 nM (Figure 1B).

**Table 1:** Mouse primer sequences used for RT-qPCR

Gene	Forward	Reverse
GAPDH	5’-ACCCAGAAGACTGTGGATGG-3’	5’-CACATTGGGGGTAGGAACAC-3’
IL-6	5’-CCGGAGAGGAGACTTCACAG-3’	5'- TCCACGATTTCCCAGAGAAC-3’
IL-1β	5’-GACCTTCCAGGATGAGGACA-3’	5’-AGGCCACAGGTATTTTGTCG-3’
TNF-α	5’-CTCTTCAAGGGACAAGGCTG-3’	5’-CGGACTCCGCAAAGTCTAAG-3’
iNOS	5’-CACCTTGGAGTTCACCCAGT-3’	5’-ACCACTCGTACTTGGGATGC-3’

**Figure 2:**
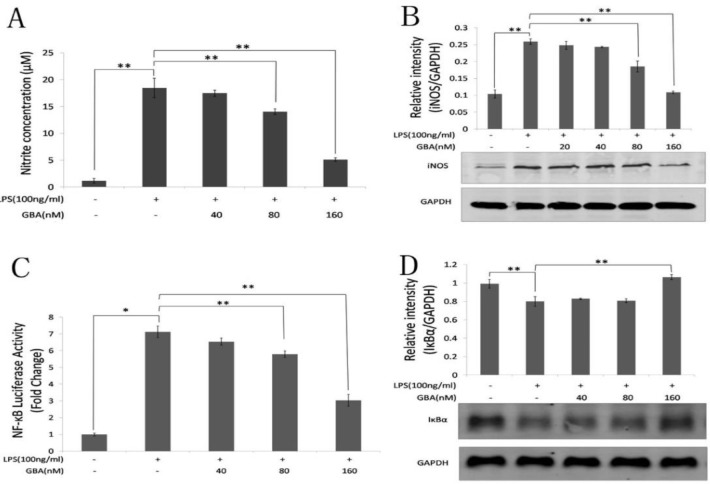
Effect of gambogic acid (GBA) on nitric oxide production, inducible nitric oxide synthase (iNOS) and nuclear factor-κB (NF-κB) pathway activation in lipopolysaccharide (LPS)-stimulated raw 264.7 cells. (A) RAW 264.7 cells were pretreated with the indicated concentration of GBA for 2 hr and stimulated with LPS (100 ng/ml) for 24 hr. Then, the nitrite level in the supernatant was measured using the Griess Reagent System. (B) Cells were pretreated with the indicated concentration of GBA for 2 hr and stimulated with LPS (100 ng/ml) for 24 hr. After cellular proteins were harvested, iNOS protein levels were measured by western blotting. (C) RAW 264.7 cells stably expressing NF-κB luciferase reporter were pretreated with different concentration of GBA for 2 hr and stimulated with LPS (100 ng/ml) for 6 hr. Then, NF-κB activation was measured by monitoring luciferase activity. (D) RAW 264.7 cells were pretreated with the indicated concentration of GBA for 2 hr and stimulated with LPS (100 ng/ml). Cellular proteins were extracted and the IκB levels were measured by western blotting. Bars represent the mean±SD of three independent experiments. * *P*<0.01 vs. normal control group; ** *P*<0.01 vs. LPS alone

**Figure 3:**
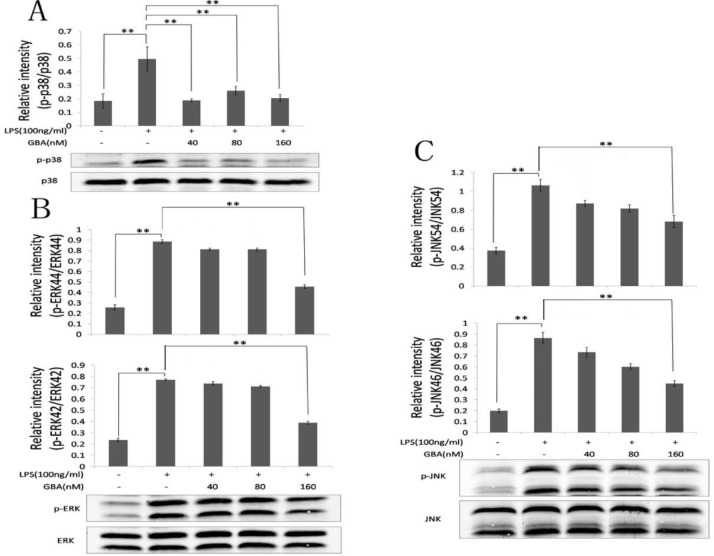
Effect of gambogic acid (GBA) on lipopolysaccharide (LPS)-induced mitogen-activated protein kinase pathways. (A) RAW 264.7 cells were pretreated with the indicated concentration of GBA for 2 hr and stimulated with LPS (100 ng/ml) for 30 min. The protein level of p-p38 and p38 were measured by western blotting. (B and C) RAW 264.7 cells were pretreated with the indicated concentration of GBA for 2 hr and stimulated with LPS (100 ng/ml) for 30 min. The protein levels of p- extracellular signal-regulated kinases (ERK), ERK, p-c-Jun N-terminal kinase (JNK) and JNK were measured by western blotting. Bars represent the mean±SD of three independent experiments. *P<0.01 vs. normal control group; ** *P*<0.01 vs. LPS alone

**Figure 4:**
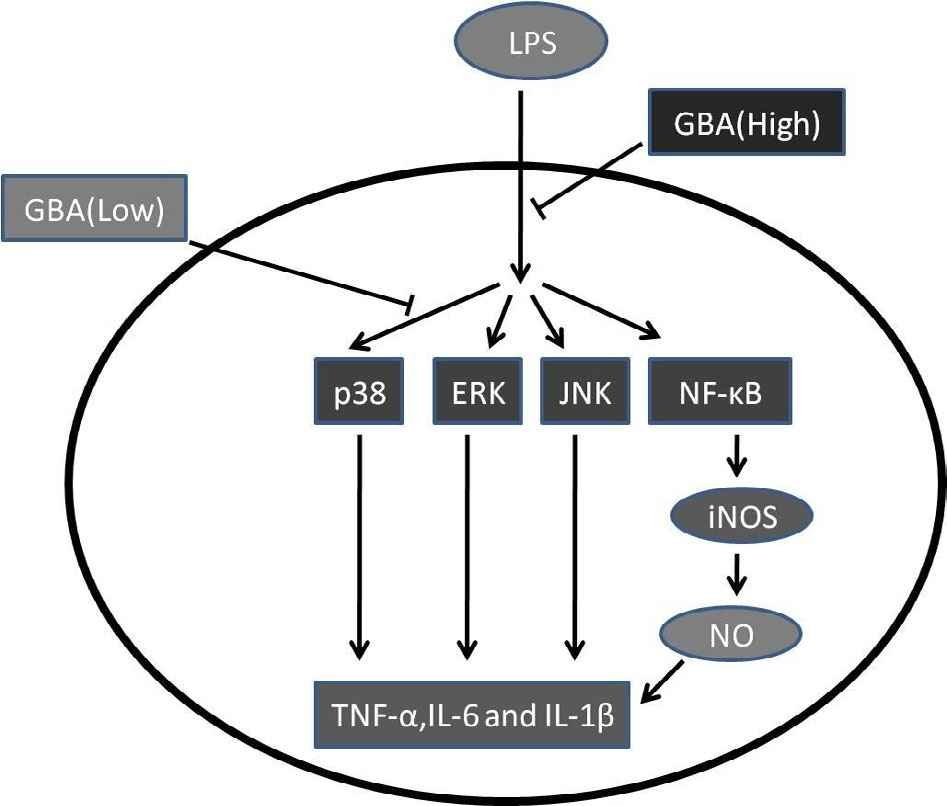
Model of the effects of gambogic acid (GBA) on lipopolysaccharide (LPS)-stimulated raw 264.7 cells. LPS triggers multiple intracellular signaling pathways, including the nuclear factor-κB (NF-κB) and mitogen-activated protein kinase (MAPK) signaling pathways. Activation of the NF- κB signaling pathway increases inducible nitric oxide synthase (iNOS), which produces nitric oxide (NO) and promotes pro-inflammatory cytokines. Pro-inflammatory genes, such as tumor necrosis factor-α (TNF-α), interleukin-6 (IL-6) and interleukin-1β (IL-1β), are upregulated upon activation of the MAPK signaling pathway. Low concentrations of GBA suppresses phosphorylation of p38; however, high concentrations of GBA has broader effects on both the MAPK and NF- κB signaling pathways, attenuating the release of pro-inflammatory factors.

***GBA effect on the release and expression of pro-inflammatory*** ***cytokines***

To investigate the effect of GBA on LPS-induced pro-inflammatory cytokine release, RAW 264.7 cells were pretreated with different concentrations of GBA for 2 hr, followed by treatment with 100 ng/ml LPS for 24 hr. As shown in Figure 1, LPS significantly increased the expression of TNF-α, IL-6 and IL-1β at secreted protein level (Figure 1C, E and G). In contrast, GBA suppressed the expression of TNF-α, IL-6 and IL-1β (Figure 1C, E and G). The 160 nM of GBA attenuated the release of TNF-α to baseline levels approximately (Figure 1C). To determine whether the amount of pro-inflammatory cytokines changed at the transcript level, we also analyzed the mRNA expression profiles of TNF-α, IL-6 and IL-1β in LPS-stimulated RAW 264.7 cells. Consistent with our ELISA results, GBA markedly attenuated LPS-induced TNF-α, IL-6 and IL-1β mRNA expression (Figure 1D, F and H). Taken together, these data suggested that GBA suppresses the production of LPS-induced pro-inflammatory cytokines at both protein and mRNA level with non-cytotoxic doses. 

***Changes in NO production, iNOS and NF-kB expression***

Nitric oxide (NO) is a key mediator of inflammation (15, 16) and has been demonstrated that it promotes the expression of pro-inflammatory mediators in LPS-stimulated RAW 264.7 cells (15, 17). Thus, we investigated the inhibitory effect of GBA on pro-inflammatory production release via decreasing the production of NO. It was found that the nitrite (NO_2_–) in the supernatant was inhibited by GBA at 80 and 160 nM (Figure 2A). Western bolt analysis confirmed that 160 nM of GBA significantly repressed the expression of iNOS (Figure 2B). Furthermore, the activation of NF-κB pathway can increase iNOS expression and NO production, which was previously reported (18). Thus, we examined the activation of the NF-κB signaling pathway after treatment of GBA. As shown in Figure 2C, LPS significantly activated NF-κB pathway in RAW 264.7 cells, which have been stably transfected with an NF-κB luciferase reporter construct. However, the activation of NF-κB pathway was suppressed by GBA at 160 nM (Figure 2C). The result of Western blot confirmed that 160 nM GBA significantly suppressed the LPS-induced degradation of IκB-α (Figure 2D). 

Interestingly, GBA did not alter the expression of NO and iNOS at low concentrations (20, 40, and 80 nM) (Figure 2A and B). Consistently, the NF-κB pathway remained active in response to low concentrations of GBA (40 nM), as demonstrated in Figure 2C and D. Therefore, high concentration of GBA (160 nM) inhibited NF-κB activation and suppressed iNOS and NO expression in LPS-stimulated RAW 264.7 cells.

***Suppression of p38 pathway***

To elucidate the mechanisms underlying the ability of low concentrations of GBA to inhibit the release of pro-inflammatory mediators, we explored the MAPK signaling pathways in LPS-stimulated RAW 264.7 cells by Western blot analysis. Interestingly, a low concentration of GBA (40 nM) significantly inhibited the phosphorylation of p38 (Figure 3A). In contrast, other MAPK signaling pathways including phosphorylation of ERK1/2 and JNK1/2 were only inhibited in response to 160 nM of GBA (Figure 3B and C). Taken together, GBA could inhibit p38 activation at low concentration (40 nM) and it displayed inhibitory effect on NF-κB, ERK and JNK activation at high concentrations (160 nM).

## Discussion

GBA is a major active component extracted from resin of *Garcinia hanburyi *and *Garcinia morella* in Southeast Asia (8, 10). Previous studies reported that GBA has anticancer effects against lung cancer (19), leukemia (9), breast cancer (20), gastric cancer (21) and hepatocarcinoma (22). Thus, GBA has been approved for use as an antitumor compound in China (8). Recently, GBA was reported to suppress inflammation in arthritis (11, 12, 23) and inhibit osteoclast formation and ovariectomy-induced osteoporosis by suppressing the JNK, p38 and AKT signaling pathway (24). This study demonstrated that GBA suppressed the production of pro-inflammatory mediator induced by LPS at both the transcriptional and translational levels. Further studies demonstrated that LPS-induced activation of p38 was inhibited by GBA with the low concentration (40 nM). In addition, high concentration (160 nM) of GBA inhibited the activation of NF-κB pathway and downregulated the expression of iNOS and NO. Furthermore, high concentration of GBA (160 nM) inhibited the activation of ERK and JNK pathways. The expression of IL-6 and IL-1β is mainly regulated by p38 pathway (25), which may account for the similar effect of GBA on the protein levels of IL-6 and IL-1β with different concentrations (Figure 1E and 1G).

Periodontitis is an inflammatory disease caused by pathogenic bacteria. Moreover, pro-inflammatory mediators, such as IL-1 and TNF-α, play a critical role in this disease (26). Recent studies also reported that the expression of IL-6 contributed to the progress of chronic periodontitis (27, 28). These observations of inflammatory reaction were consistent with our results of LPS-induced RAW 264.7 cells. Activation of p38 signaling pathway is crucial for gene expression of pro-inflammatory mediators (29-31). Previous studies demonstrated that p38 inhibitors significantly decrease both the protein and mRNA levels of pro-inflammatory mediators (32). Kotlyarov *et al*. reported that knockout of MAPKAP kinase 2 (MK2), a downstream target of p38, suppressed LPS-induced overexpression of TNF-α, IL-6 and IL-1β, which demonstrated the importance of the p38 signaling pathway for pro-inflammatory factor production (33). Here, we reported that low concentrations of GBA (40 nM) markedly inhibit the phosphorylation of p38, which explains how GBA inhibits the release of TNF-α, IL-6 and IL-1β.

Although the NF-κB signaling pathway and other two MAPK pathways were not significantly inhibited by low concentration of GBA, other studies demonstrated that GBA could inhibit the activation of ERK signaling and JNK signaling pathway in pancreatic cancer cells (34) and osteoclast formation (24), respectively. The inhibitory effect of GBA on NF-κB signaling pathway could also be found in lung metastasis (35). Furthermore, a previous report indicated that GBA (500 nM) inhibited the release of pro-inflammatory mediators by blocking the NF-κB signaling pathway (36). Consistent with their results, we observed that high concentration of GBA (160 nM) inhibited the NF-κB signaling pathway and suppressed the expression of iNOS and NO. Moreover, high concentrations of GBA (160 nM) suppressed the activation of ERK, JNK and p38 pathways. Thus GBA elicited inhibitory effect on various signaling pathways, including the NF-κB and MAPK pathways. 

To identify natural compounds that prevent the LPS-induced inflammatory response, we identified GBA as an antagonist of LPS-induced pro-inflammatory cytokines. Interestingly, a low concentration (40 nM and 80 nM) of GBA significantly suppressed the p38 pathway, and a high concentration (160 nM) of GBA only suppressed the NF-κB and other MAPK pathways (ERK and JNK). Taken together, our data demonstrates that GBA inhibits the release of TNF-α, IL-1β and IL-6 mainly through suppression of the P38 pathway (Figure 4).

## Conclusion

This study demonstrated that GBA is a potential pro-inflammatory inhibitor due to the suppression of p38 pathway. In addition, the inhibition of NF-κB and other MAPK signaling pathways at high concentrations of GBA (160 nM) has an additive inhibitory effect on pro-inflammatory factor release. Given the importance of pro-inflammatory factor production in periodontitis and the potent inhibitory effect of GBA on inflammation, GBA is a promising compound for the treatment of periodontitis disease. Further studies are planned to test the efficacy of GBA *in vivo*.

## Conflicts of Interest

The authors report no declarations of interest.
